# Scrutinize of healthy school canteen policy in Iran’s primary schools: a mixed method study

**DOI:** 10.1186/s12889-021-11587-x

**Published:** 2021-08-18

**Authors:** Mina Babashahi, Nasrin Omidvar, Hassan Joulaei, Azizollaah Zargaraan, Farid Zayeri, Elnaz Veisi, Azam Doustmohammadian, Roya Kelishadi

**Affiliations:** 1 Department of Community Nutrition, National Nutrition and Food Technology Research Institute, Faculty of Nutrition Sciences and Food Technology, Shahid Beheshti University of Medical Sciences, Tehran, Iran; 2 Health Policy Research Center, Institute of Health, Shiraz University of Medical Sciences, Shiraz, Iran; 3 Department of Food and Nutrition Policy and Planning Research, National Nutrition and Food Technology Research Institute, Faculty of Nutrition Sciences and Food Technology, Shahid Beheshti University of Medical Sciences, Tehran, Iran; 4 Proteomics Research Center and Department of Biostatistics, Faculty of Allied Medical Sciences, Shahid Beheshti University of Medical Sciences, Tehran, Iran; 5 Gastrointestinal and Liver Diseases Research Center, Iran University of Medical Sciences, Tehran, Iran; 6 Department of Pediatrics, Child Growth and Development Research Center, Research Institute for Primordial Prevention of Non-Communicable Disease, Isfahan University of Medical Sciences, Isfahan, Iran

**Keywords:** Policy analysis, Primary schools, Nutrition policy, Food policy, School health services, Children, Iran

## Abstract

**Background:**

Schools provide an opportunity for developing strategies to create healthy food environments for children. The present study aimed to analyze the Healthy School Canteen (HSC) policy and identify challenges of its implementation to improve the school food environment in Iran.

**Methods:**

This mixed method study included two qualitative and quantitative phases. In the qualitative phase, triangulation approach was applied by using semi-structured interviews with key informants, documents review and direct observation. Data content analysis was conducted through policy analysis triangle framework. In the quantitative phase, food items available in 64 canteens of primary schools of Tehran province were gathered. The food’s nutrient data were evaluated using their nutrition facts label. The number and proportion of foods that met the criteria based on Iran’s HSC guideline and the World Health Organization nutrient profile model for the Eastern Mediterranean Region (WHO-EMR) were determined.

**Results:**

The main contextual factors that affected adoption of HSC policy included health (nutritional transition, high prevalence of non-communicable diseases and unhealthy food environment in and around the schools), political (upstream supportive policies and joint memorandums about health children between the Ministry of Health and Medical Education and Ministry of Education), structural (the lack of unified stewardship, inadequate human resource capacity, poor inter-sectional cooperation), economic (school financial problems, poor fiscal supportive of food policies), and socio-cultural (mothers working outside the home, the role of children’s peer group, low nutrition knowledge of school principals) factors. Assessment of the school canteens showed that a large proportion of available foods did not comply with the national guidelines (54.7 ± 2.54%) and WHO-EMR model (85.6 ± 2.34%). The main reasons identified for incomplete implementation of the policy were inadequate physical and economic infrastructure to set up standard school canteens, lack of scientific criteria for food categorization, poor monitoring, high price of healthy foods, and conflict of interest among the actors.

**Conclusion:**

The majority of foods and beverages available in the school canteens did not comply with national and regional standards. Iran HSC policy needs to be improved by using an evidence-based approach and active interaction between all key actors.

**Supplementary Information:**

The online version contains supplementary material available at 10.1186/s12889-021-11587-x.

## Background

Childhood is a critical period to form lifelong dietary behaviors which influence future risk of obesity and diet-related non-communicable diseases (NCDs) [1, 2]. Despite this fact, the prevalence of obesity and NCDs in children and adolescents are increasing in both developed and developing countries and has become a major health challenge [3]. Approximately 23% of children are overweight or obese in developed countries [4]. Previous studies indicate that about 30 and 15% of school age-children in North America are overweight and obese, respectively [5]. Also, prevalence of overweight or obesity was estimated 20% in school age-children of European countries [5]. In the developing countries, the prevalence of overweight and obesity in children has increased from around 8 to 13% during 1980 to 2013 [4]. Over the last decades, Iran as a middle-income developing country has encountered a rise in obesity, metabolic syndrome and diabetes among children and adolescents [6–8]. The prevalence of overweight and obesity in Iranian population aged less than 18 years was estimated 5.5% (95% CI,5.1–6.0) and 15.1% (95% CI, 13.5–17), respectively from 2000 to 2013. Also, a nationwide study in 2015, showed that over 20% of students aged 7–18 years had excess weight and abdominal obesity [9].

Food environment is likely more effective to the increasing epidemic of obesity and chronic diseases than individual factors e.g. knowledge, skills, and incentive [10]. Food environment comprises the “*range of foods that can be accessed in the context where people live and can enable or restrict healthy dietary choices*” [11] and include aspects such as food composition, safety, promotion, labelling, prices, trade policies, and provision in different settings, including schools [12]. Over the past decades, major changes in the food environments have been driven by technological advances, urbanization, food and agricultural policies, and economic, social, and lifestyle changes [13]. More processed and convenience foods are now readily available and accessible in multiple settings throughout the day in larger portion sizes and at relatively low prices [14]. Children often have less control over their food environment [15]. Living in unhealthy food environments can easily encourage them to low quality food patterns by increasing and facilitating access to unhealthy food choices. Therefore, unhealthy food environments are considered risk factors affecting malnutrition in children and drive the increase in the prevalence of obesity and NCDs [11, 15].

Children spend many hours at school each day [16]; therefore, schools are considered as an important setting to promote healthy behaviors among school age children [17, 18]. Global recommendations for the prevention of NCDs, including the Global Action Plan for NCDs [19] and the Rome Declaration on Nutrition and Framework for Action (2014) [20], have consistently called for heightened action on nutrition in school settings. The World Health Organization (WHO)'s ‘Report of the commission on ending childhood obesity’ (2016) recommends establishing standards for meal provision to meet nutrition guidelines, eliminating unhealthy foods from the school environment and establishing mechanisms to safeguard public health from conflicts of interest [21]. School-based interventions promoting consumption of healthy food and non-alcoholic beverages are frequently reported to be among the most cost-effective diet-related approaches to NCDs prevention [22, 23]. In this regard, there are several policies/programs that directly or indirectly affect the food environment of schools in Iran, e.g., banning food marketing and advertising of unhealthy food and sweetened beverages in schools, healthy school canteen policy, health-promoting school program, the IRAN-ending childhood obesity program, weight and obesity control in students (Kouch) [24]. These programs have helped to restrict unhealthy food advertisements in the school environment, control the food items in the school canteens, increase the nutritional awareness of children, parents, and school staff, increase physical activities, monitor nationwide children’s weight, and refer malnourished children to nutritionists [24].

School canteen is an important component of school food environment that can effect children’s eating and dietary practices [25], and can contribute to the high intake of energy-dense, nutrient-poor (EDNP) foods [26, 27]. It has been shown that for creating healthy environments for children and reducing childhood obesity, improving nutritional quality of the food offered in schools is necessary [28–30]. Nutrition policies governing the availability of foods in schools have been recommended by the WHO to improve child nutrition [31]. Such policies offer an opportunity to ensure that the foods made available to students in schools, comply with dietary guidelines and often restrict sales of specific food and beverage items (e.g., soft drinks) or set nutrition standards to determine what foods and beverages can be sold in schools [32, 33]. Systematic reviews on the effectiveness of policies restricting the availability of unhealthy foods have consistently shown positive effects on children’s diet [34, 35]. Despite the popularity of school nutrition policies, they are poorly implemented [26]. For example, in Australia, only 5–35% of schools in most states obey from government healthy canteen policies with minimal improvements with increasing duration time of policy implementation [18, 36, 37]. Thus, implementation of recommended policies for healthy food provision in schools has been inadequate and inconsistent globally [38].

In Iran, since 2014, the Ministry of Health and Medical Education (MoHME) have formulated the national guidelines for Healthy School Canteen (HSC) in collaboration with the Ministry of Education (MoE). Based on this guideline, all schools in Iran have to comply and provide healthier food and drink choices for school canteens [39]. Evidence from Iran shows that foods sold in schools include items that their sales have been banned by the HSC policy, e.g. chips, cookies, crackers, ice cream, fried foods, sugary drinks, hamburgers, pizza, and confectionary [40, 41]. Therefore, considering the association between school food environment and eating behaviors of children [40–43], providing a more appropriate food environment through strengthening such policies is a priority.

This study aimed to analyze HSC policy through the policy triangle framework focusing on context, content, policy process, and actors and to evaluate its outcome.

## Methods

The present study, using a mixed method approach consisted of two phases. In phase one (qualitative part), policy-making process for improvement of the food environment through HSCs in Iran was clarified and analyzed through a retrospective policy analysis. In the second phase (quantitative approach), in order to evaluate the policy outcome, food items available in primary schools’ canteens in Tehran province were evaluated. The study was performed from November 2018 to October 2019.

### Phase 1: a retrospective policy analysis

#### Design

This phase included a cross-sectional study, aimed to analyze policies and regulation related to school food environment in Iran through content analysis of available documents and in-depth interview with key informants.

#### Data collection

A review of the literature and documents was conducted for tracking of HSC policy in Iran. Key websites from MoHME (https://behdasht.gov.ir), MoE (https://www.medu.ir) and Islamic Parliament Research Center (http://rc.majlis.ir/fa/law), WHO (https://www.who.int), Google scholar and Scopus were searched for any document, article, or report related to the process of HSC policy formation, executive instructions, and key functions of each organization/office that collaborated in policy implementation, from January 2000 to September 2019. In addition, relevant minutes, guidelines, and documents were made available by the experts from the offices of the MoHME and MoE.

Interviews were conducted with key informants selected through purposeful and snowball sampling from February to October 2019. The first author performed semi-structured in-depth interviews using an interview guide that was developed for this study (Additional file 1). Prior to the face to face interview, the questionnaire was provided to the interviewees via email. Through the interview process, interviewer were also asked probing or follow-up questions on the basis of interviewees’ answers to previous questions [44]. The duration of interviews ranged from 30 to 40 min. All interviews were done in the workplace of interviewees, recorded with their permission and transcribed verbatim by a single interviewer. Data reached saturation at the 44th interview, and four more interviews were performed to ensure it. Interview respondents included information-rich individuals on HSC policy at three levels, including: 1) National level informant in MOHME and MoE (*n* = 13), 2) Provincial level actors in the departments of education and deputy of health in the medical sciences universities (*n* = 7), and 3) local level actors and stakeholders from comprehensive community health centers, health houses, school officials, canteens managers, food producers and food distributors for school canteens, as well as students’ parents (*n* = 28).

#### Data analysis

The policy triangle framework adapted from Walt and Gilson (1994) and stages heuristic model [45, 46] were used for the policy analysis framework. The deductive content analysis was done concurrently with data collection. The transcriptions were carefully read several times to achieve main concepts in open coding. Subsequently, subthemes and themes were extracted based on the study objectives. Data and codes management was performed using MAXQDA software (Version 10, Berlin, Germany) [47].

#### Data trustworthiness

Guba and Lincoln’s criteria including credibility, dependability, transferability, and confirmability were used to assess the trustworthiness of the data [48]. Triangulation approach was applied to strengthen credibility [49] by using data gathering from multiple sources, including document reviews, in-depth semi-structured interviews with a range of different informants, and observations. Besides, experts/ member checking of the interpretations and analysis was done to boost credibility. To improve data dependability, 30% of documents and the transcribed interviews were randomly selected and reanalyzed by a second researcher (AD) independently.

Also, dependability was fulfilled through in-depth discussions with experts and a review by the participants and other researchers. The transferability was addressed by explicating and providing an accurate description of interviewee characteristics, data collection, and analysis process in all research stages. To ensure of the data’s confirmability, the main researcher kept all documents and recorded data to re-evaluate them and remove any inconsistencies between interpretations.

The design and reporting of this phase of the study was done based on Consolidated criteria for Reporting Qualitative research (COREQ) [50].

### Phase 2: assessing primary schools’ canteens

#### Design

This phase comprised of a descriptive, cross-sectional study, carried out in 64 primary schools in Tehran province from November 2018 to March 2019.

#### Selection of models/guidelines

Two models/guidelines were selected for evaluating food items available in school canteens. Iran’s HSC guideline [39] was used as the national guideline and nutrient profiling model of the World Health Organization in Eastern Mediterranean Region (WHO-EMR) [51] was selected as the regional standard. The unhealthy snacks are implicitly defined in the national bylaw as high-calorie foods with high fat and/or salt content, low nutrient density and low food safety, and, while healthy snacks are defined as dairy products, nuts, vegetables, fruits, and other foods with low salt and sugar content [39]. WHO-EMR nutrient profile model is a guide for policy-makers, developed in EMR region and provides a single systematic method of regulation in the provision of food to public institutions, for example schools [51]. This model consists of 18 food categories, with some subcategories [51]. Six food categories are completely “not permitted” for marketing to children because of their high content of sugar, fat and salt [51]. For determining whether a food product in other categories is marketable to children, the energy density and nutrient content must be assessed [51].

#### School sampling

Schools were selected using a multistage systematic cluster sampling framework. In the first stage, among the counties of Tehran province, Tehran city, and five counties from five educational zones of counties around Tehran city (Shahriyar, Robat Karim, Kahrizak, Pakdasht and Damavand) were selected. In Tehran city, the department of Education includes 19 educational districts and classifies them into 3 socioeconomic levels: affluent, semi affluent, and deprived. Therefore, three districts from each of the three socioeconomic categories were chosen based on the geographical location, i.e. one district from each of the west, east, and central districts of each category.

In the second stage, a list of primary schools that had a canteen was collected from the respective department of education of each included district or county. There was a food canteen in almost one-third of schools (36%). Then, schools were selected from the proportional strata defined by gender and type of school (public/private).

#### Data collection

A researcher-made questionnaire was used to record general school characteristics through interviewing school principals (Additional file 2). In each school, characteristics of packaged industrial foods available at the canteen were recorded by taking photos of all sides of the packages. The information collected from each package included product information (e.g., product name, company, and brand), net weight, and nutrition facts table information. Only one package size per food product was captured, but all flavors and varieties were collected. In case of traditional or homemade foods (e.g., Ash Reshteh, Halim and Kookoo-ye Sabzi sandwich), recipe analysis was done to determine their nutrient content. In this regard, we collected the type and amount of ingredients in these foods by interviewing food preparation staff in the canteen. Nutrients and energy content of these food items were determined by the Nutritionist IV software, version 7.0 (N-Squared computing, USA), in which Iranian food composition table is also added. Compliance with the guidelines was checked through comparing nutrient content of food items (specifically total sugar, added sugars, non-sugar sweeteners, total fat, saturated fatty acid, salt and energy) with the WHO-EMR model and Iran HSC guideline.

#### Data analysis

Food items in the school canteens were classified into ‘permitted’ or ‘not permitted’ based on the WHO-EMR model and HSC guideline of Iran. Descriptive statistics and appropriate statistical tests, including independent samples t-test, paired samples t-test, and one-way analysis of variance were used for data analyzing by using SPSS, version 16 (SPSS Inc., Chicago, IL, USA). The level of significance was set at *p* < 0.05.

#### Ethical considerations

The ethical committee of the National Nutrition and Food Technology Research Institute approved the protocol of present study (No IR.SBMU.NNFTRI.REC.1397.035).

## Results

The findings of the qualitative part of the study are categorized based on the policy analysis triangle framework, i.e., policy content, context, policy-making process, and actors, as follows:

### Policy content

This bylaw specifically addresses the school canteens, and reflects policymakers’ attention to the importance of school food environment. The goals of this bylaw are increasing access to healthy snacks, preventing the supply of foods with low nutritional value, modifying children’s nutrition patterns, providing a portion of required energy (300 kcal per day in snacks at school), protein and necessary nutrients, and maintaining and promoting health and prevention of non-communicable diseases. To ensure effective implementation, standardizing and effectively monitoring the canteen, and reinforcing nutritional knowledge among students have been emphasized.

One of the most important strengths of this bylaw is being based on the collaboration of two ministries of Education and Health and defining their responsibilities to help improve the health status of the school’s canteens. Also, the bylaw is providing a list of permitted and not permitted food items in order to set a standard for food items offered at school; this list is required to be installed in schools in a suitable place and in the view of students and school staff [39].

On the other hand, there are a number of weaknesses in the content of this bylaw as follows:

- Lack of clear and measureable criteria to determine permitted and not permitted food items.

There is no clear, definite and scientific criterion for distinguishing between permitted and not permitted food items. The regulation divides food items into two groups based on their safety and health characteristics and defines healthy foods as: *“Healthy or safe food are those prepared from healthy and safe raw materials, and are free from contamination and harmful ingredients.”* [39]. This definition does not provide a threshold or cut off for the mentioned indicators. Therefore, it makes objective judgment about the food items impossible.

- Inadequate diversity in food list provided, and limited attention to local food culture and availability.

One of the most important shortcomings of the bylaw that was also mentioned in the interviews is the low variety in the list of permitted food items.

*“Our request from the industry is to make something [Food product] that has both nutritional value and beautiful and attractive package. … We don’t have a variety of products that can be offered in the school canteen, and that’s a weakness.”* (A representative from Community Nutrition Office in MoHME).

Therefore, it is necessary to include more diverse food items in the permitted foods list according to local conditions and individuals’ food habits in different regions of the country.

-Not providing a solution to establish a standard and healthy food canteen in schools.

One of the major weaknesses of the HSC bylaw is little attention being paid to the condition of most school canteens in Iran. According to the interviews, most school canteens do not have proper building and physical condition. Therefore, it seems necessary to define specific criteria for a hygienic school canteen and express strategies to improve present canteens. Most interviewees mentioned school budget shortages as a limitation to improve canteen’s physical condition.

*"... A large number of schools in the country, even in Tehran city, have almost worn-out buildings. The buildings are very old. A lot of money must be provided to repair and renovate school buildings and their physical spaces*." (A representative of Student Organization)

Therefore, funding strategies is another point that should have been considered in the content of the policy.

-Ambiguity in the role(s) of program stakeholders.

Despite having a wide range of actors and stakeholders related to this policy, reviewing the documents shows that the assigned roles in bylaw have been limited to the MoHME and MoE, while the expectations of stakeholders from each other have not been clearly defined.

"*The content of the program does not specify who should participate in the program. For example, food industry, as a private sector, plays an important role in the development of healthy eating, but its role in the program is not clear."* (A representative of the Health office in the Department of Education)

Thus, more transparency with regard to specific roles of these two and many other stakeholders, including food industry, parents and students is needed.

-Unclear strategic and operational planning.

One of the main weaknesses of the bylaw’s content is the lack of strategic planning along with a clear, comprehensive and step-by-step national operational plan. However, the executive guideline is annually provided by the MoHME and MoE to the relevant departments throughout the country, but the annual actions and short-term goals are not clearly described in detail.

-Defect to evaluate the program.

There is unclear mechanism for comprehensive monitoring and evaluation of the program. A review of the policy documents shows that although internal and external audits have been defined to monitor the program, no comprehensive evaluation mechanisms that include an assessment of the program’s inputs, process, outputs, impacts and outcomes at various national, province, county, and school levels has been defined.

### Policy context

Five main themes were identified as the main contextual factors lead to the formulation and implementation of the HSC program, as follows:

-Health factors.

Following socio-demographic changes and urbanization in Iran, the nutrition transition is taking place rapidly in recent decades [7]. Healthy traditional diets have been shifting toward less healthy dietary habits such as high consumption of processed/ultra-processed foods and high-calorie, low-nutrient-density food. The emergence of a high prevalence of overweight, obesity and other metabolic change in Iranian children is a serious health problem [8]. For improving children’s health, schools are proper environments for health policy interventions. According to the informants at the MoHME, high availability and accessibility of unhealthy foods in the schools or food stores around schools are noted as a major challenge to ensure children healthy food choices during school hours.

-Political factors.

Upstream documents in Iran, e.g. Fourth National Economic, Social and Cultural Development Plan [52] was a strong supportive policy for the healthy nutrition of students. In one of its articles, allocating resources to student snacks has been clearly addressed. Also, in the National Nutrition and Food Security Document [53], providing safe and healthy food in public places for children has been stated as one of the major priorities. Moreover, there has been a long history of inter-organizational cooperation between the MoHME and MoE for children’s health in schools which has led to several joint memorandums, including HSC in 2014.

-Structural factors.

Existence of various executive offices in the MoHME and MoE related to school health policies, and relatively little interaction between them can lead to inadequate use of available human and financial resources, parallel work and hinder reaching the goals of the HSC policy. In addition, lack of unified and stable stewardship has sometimes caused disagreement between the decision-making centers of the two ministries in some cases about health program in schools. Centralized regulations without attention to regional or local needs and limited participation of school principal in decision-making committees regarding student nutrition are important reasons behind incomplete implementation of the program in schools. Moreover, according to informants at the Student Organization, many schools have old buildings and do not have enough physical space to establish a healthy canteen.

-Economic factors.

In the context of international economic sanctions on Iran, food prices are rising sharply [54]. Many experts believed that the financial profitability of unhealthy food products encourages canteen owners to sell more of them to children. Unhealthy foods are sold more because of their lower price compared to healthy foods and children preference of their savory taste. Many experts agreed low budget and financial resources are allocated to school affairs. In some schools, staffs claimed that school canteens can be a source of income; therefore, some principals have partnered with canteen managers to sell unhealthy food in order to cover part of the school expenses.

-Socio-cultural factors.

Many interviewed mothers who were employed stated that they could not provide a suitable snack for their children because they did not have enough time for preparing it. Moreover, their children are influenced by their peers and classmates to purchase foods from the school canteen. Also, the abundance of unhealthy food advertisements in the outdoor environment, television and social media encourages their children to consume them.

Some informants at MoHME expressed that limited nutritional knowledge of some school principals and most canteen managers is one of the main obstacles to stop selling unhealthy food in schools and compliance in implementation of HSC policy.

### Policy actors

The two main actors in different stages of the policy process of the HSC policy are the MoHME and MoE. The offices of community nutrition and environmental health in the MoHME, and health office in MoE have key roles. The office of community nutrition in the MoHME have the main responsibility for designing school nutrition guidelines and identifying permitted and not permitted food list. Supervision of the establishment and quality management of the healthy school canteens has been assigned to the Student Organization in the MoE.

Some of the interviewees were dissatisfied with the performance of the Student Organization and stated that they had a profit-based approach and considered the regulations as a mean for the financial profit of this organization.

*"... The student organization is also involved in issuing license for school canteens. According to their instructions, they should receive some money from school principals to issue a license of healthy canteen. Some principals do not declare that their school has a canteen because they do not want to pay that money and this is a damage* [to policy implementation]*."* (A representative of Health office in the Department of Education).

It is necessary to have effective communication between key actors to justify policy goals and remove executive barriers.

In the present study, most canteen managers were janitors (76.56%), followed by other school staff (12.5%), students (6.25%), and in some cases personals employed by private sector (4.68%). School principal is responsible for the status of the school canteen. According to interviews, in some schools, implementation of this bylaw creates conflicts of interest for the principals. On one hand, the supply of healthy food items helps to improve students’ nutrition and on the other hand, if the canteen is not financially profitable, principals cannot afford to compensate expenses through the school’s budget and may fall under economic pressure. As a result, some principles are not motivated enough to enforce regulations and resist, while as the leader of the program in the school, can play a valuable role in conveying the health messages of the canteen. In addition, in some schools, canteen managers do not fully comply with the rules due to the financial benefits of selling unhealthy food products.

In eight schools (12.5%), students’ mothers cooperated in preparing cooked foods. Based on school officials, collaboration of mothers has a positive effect in health status of canteens. After all, the food industry can help improve the health of the children’s food environment by producing varied healthy food products, but according to the interviews and review of the documents, they have not been properly involved in this policy.

### Policy process

**Fig. 1:**
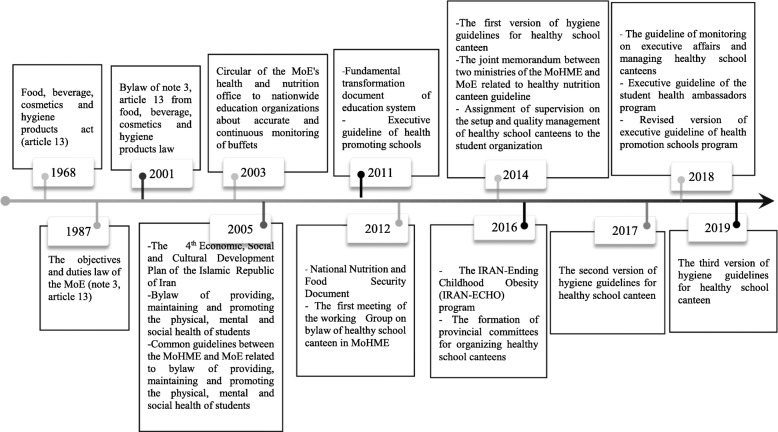
A historical chronology for the healthy nutrition canteen policy making in Iran (1966–2019). MoHME: The Iranian Ministry of Health and Medical Education; MoE: The Ministry of Education

Although the initial attention on healthy food environment goes back to 1968 through article 13 of food, beverage, cosmetics and hygiene products act [55], the idea of healthy schools canteen was started as part of the general school health programs in 2013. The first HSC guideline was formulated in the same year and has been revised twice since then in 2017 and 2019. This bylaw has also been used in two other programs, including the IRAN-Ending Childhood Obesity (IRAN-ECHO) program [56] and health promoting schools program [57] to improve schools food environment. The trend and milestones of HSC policy in Iran is shown in Fig. 1.

The heuristic stages method was used for analyzing the policy-making process [46]. This model contains four stages of agenda-setting, policy formulation, policy implementation, and policy evaluation. The analysis of the policy making process with regard to healthy school food canteen in Iran follows:

### Agenda setting

Most interviewees believed that from decades ago, the hygienic status of school canteens was the main concern of both the MoHME and MoE; however, the recent alarming increase in childhood overweight and obesity, along with evidence on high consumption of unhealthy foods by children (CASPIAN study) and access to unhealthy food in schools made the healthiness of school food environment a major priority. Increasing inter-sectoral communications between MoHME and MoE for joint programs such as Health Promoting Schools Program or Executive Regulations for Providing, Maintaining and Promoting Physical, Mental and Social Health of Students provided a good background for cooperation of the two ministries. The result was a joint working group for the formulation of a bylaw on healthy canteens in 2012 and holding several meetings in the MoHME.

### Policy formulation

The first bylaw was signed by the two ministers in 2014 in the form of a joint memorandum of understanding. However, the policy formulation approach was not evidence-based and did not follow a valid scientific process for settling different stages of the bylaw, including the criteria to define the list of permitted and unauthorized food items. Therefore, it was largely based on the previous relevant experiences of policymakers and experts from the Offices of Community Nutrition and Health Environment in the MoHME and Health office of the MoE [52]. However, except for the two mentioned offices, no intra- and inter-sectoral partnership was involved in policy formulation. This haste in design and expansion of the program with a top-down approach has caused little attention being paid to the existing capacity, constraints, and infrastructure of the school canteens, and has led the program not completely being implemented.

### Policy implementation

After notifying school principals regarding the regulations, they are required to obtain a license from the Student Organization to set up a canteen to supply food items according to the permitted foods list recommended by the HSC bylaw. Various supervisory groups from the Student Organization, the Office of Health in the Department of Education, the Nutrition and Environmental Health Unit of Community Health centers, monitor the implementation process of the policy. Several committees at the MoHME or MoH and provincial committees are set up to review and coordinate policy implementation. Although it has been more than four years since the launch of this program, its implementation has always faced challenges, the most important of which are listed in Table 1.

**Table 1:** Implementation challenges of healthy school canteens policy in Iran

Themes	Subthemes	Code
Inappropriate management approach	Top-down management approach	Poor and passive participation of the school principal
		An inspection-based approach in experts with poor support for training and counseling to improve policy implementation
	Retardant requirements	Receipt of fees for issuing a card health card for canteen worker
		Receipt of fees from the school administrators for the issuance of a nutrition canteen license by the Student Organization
		Mandatory prior notification of the presence of nutrition experts in the school
Weakness in program coordination	Poor intra-sectoral cooperation and coordination	Poor involvement of all stakeholders/ actors invited to the program committees
		Insufficient knowledge of the school administrators about the program and leaving the responsibility entirely to the healthcare interface or health coach
		Low level of cooperation and supervision of school principals in offering not permitted food products through canteens
	Poor inter-sectoral cooperation and coordination	Poor cooperation with producers and distributors of food products
		Limited cooperation of the Broadcasting Organization
		Poor coordination with students’ parents
	Poor advocacy	Lack of parental advocacy for monitoring of canteens
		Inadequate advocacy for the Broadcasting Organization to create a nutritional culture and introduce the program
Weakness in resource management	Bottlenecks of providing and allocating financial resources	Insufficient budget of responsible organizations
		Coverage of part of the school’s expenses from the sale of unhealthy food
	Weak management of human resources	Inadequate manpower
		Inappropriate motivational system
	Inadequate and insufficient facilities and equipments	Inadequate physical space of the food canteen
		Insufficient and inappropriate equipment(s)

**Fig. 2:**
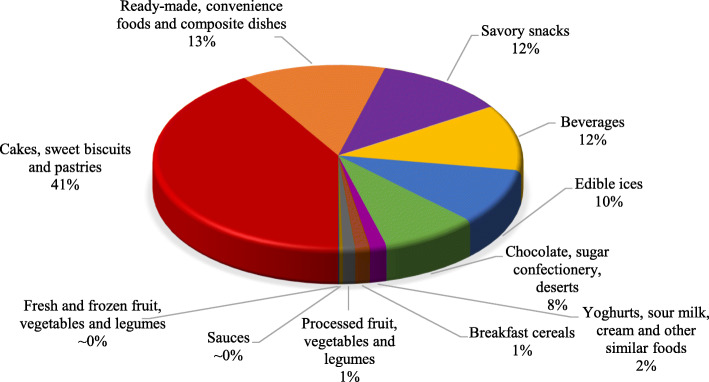
Proportion of different food categories available in the canteen schools

The availability of categories of food items in the school canteens are presented in Fig. 2. As shown, the category of cakes, sweet biscuits and pastries has the largest share.

The degree of strictness, as shown by the proportion of food items classified as permitted to be accessed in school canteens, varied considerably between the different WHO-EMR model and healthy nutrition canteen guideline of Iran (Table 2-4). WHO-EMR model is stricter and overall only 14.4% of food items in the school canteens complied with it (Table 2). There was a significant difference between the proportion of total permitted and not permitted food items in the schools based on both criteria (Table 2).

**Table 2:** Total food items in school canteens (*n* = 64) assessed as ‘permitted’ or ‘not permitted’ based on the WHO-EMR model and healthy school canteen guidelines of Iran

Criterion	The mean percentage of food items in the canteens (SE)		*P-value*
	Permitted	Not permitted	
WHO-EMR model	14.4 (2.34)	85.6 (2.34)	> 0.001**
National guideline	45.3 (2.54)	54.7 (2.54)	0. 01**
*P-value*	> 0.001*	> 0.001*	

*Statistically significant at 5% level using the paired samples t-test

**Statistically significant at 5% level using the independent samples t-test

**Table 3:** Frequency distribution of permitted and not permitted food items existing in the canteens of primary schools in Tehran province (n = 64) by the WHO-EMR model and healthy school canteen guidelines of Iran

Food categories based on the WHO-EMR model	Foods permitted by the WHO-EMR model, n (%)	Foods not permitted by the WHO-EMR model, n (%)	Foods permitted by the national guideline, n (%)	Foods not permitted by the national guideline, n (%)
1. Chocolate, sugar confectionery, desserts	–	(9.2) 86	–	(13.7) 86
2. Savory snacks	8 (5.9)	(12.1) 113	(5.2) 23	(15.6) 98
3. Beverages	(14.0) 19	(15.7) 147	(9.4) 42	(19.7) 124
4. Edible ices	–	(10.8) 101	–	(16.1) 101
5. Breakfast cereals	–	(1.4) 13	–	(2.1) 13
6. Cakes, sweet biscuits and pastries	–	(45.2) 424	(54.6) 243	(28.8) 181
7. Yoghurts, sour milk, cream and similar foods	–	(1.6) 15	(3.4) 15	–
8. Ready-made and convenience foods and composite dishes	(77.9) 106	(2.8) 26	(24.7) 110	(3.5) 22
9. Fresh and frozen fruit, vegetables and legumes	(0.7) 1	(0.0)0	(0.2) 1	–
10. Processed fruit, vegetables and legumes	(1.5) 2	(0.9) 9	(2.5) 11	–
11. Sauces	–	(0.3) 3	–	(0.5) 3

**Table 4:** Frequency distribution of permitted and not permitted food items existing in the canteens of primary schools in Tehran province (n = 64) by the WHO-EMR model and healthy school canteen guidelines of Iran

Variable	Number of schools	Mean of permitted food items percentage (SE)		Mean of not permitted food items percentage (SE)	
		Based on the WHO-EMR model	Based on the national guideline	Based on the WHO-EMR model	Based on the national guideline
**School type**					
Public	26	13.63 (3.08)	41.78 (2.69)	86.36 (3.08)	58.22 (2.69)
Private	38	15.40 (3.65)	50.42 (4.75)	84.59 (3.65)	45.57 (4.75)
***P-value***		0.838	0.138	0.838	0.138
**Student gender in schools**					
Girl	29	15.59 (3.78)	50.23 (3.65)	84.40 (3.78)	49.67 (3.66)
Boy	33	12.62 (2.89)	42.54 (3.37)	87.38 (2.88)	57.45 (3.37)
***P-value***		0.513	0.138	0.513	0.138
**School area**					
Urban	53	16.88 (2.69)	46.24 (2.92)	83.12 (2.69)	53.76 (2.92)
Rural	11	2.20 (1.30)	40.73 (4.42)	97.80 (1.30)	59.26 (4.46)
***P-value***		0.004*	0.499	0.004*	0.499
**School region**					
Tehran city districts					
Affluent	12	11.57 (3.43)	38.51 (5.59)	88.42 (3.34)	61.49 (5.59)
Semi-affluent	11	23.72 (5.38)	50.61 (6.48)	76.27 (5.39)	49.38 (6.48)
Deprived	12	15.37 (8.01)	50.99 (7.40)	84.62 (8.01)	49.01 (7.40)
Shahriyar county	6	10.63 (9.00)	43.01 (5.80)	89.36 (9.00)	59.99 (5.81)
Robat Karim county	6	11.34 (5.02)	42.66 (6.51)	88.66 (5.02)	57.34 (6.51)
Kahrizak county	6	17.33 (7.79)	59.63 (6.49)	82.67 (7.79)	40.36 (6.49)
Pakdasht county	5	5.51 (3.37)	40.51 (5.88)	94.85 (3.37)	59.48 (5.88)
Damavand county	6	12.14 (7.91)	32.23 (8.29)	87.86 (7.9)	66.76 (8.29)
***P-value***		0.470	0.355	0.470	0.355

*Statistically significant at p < 0.05 using independent t test

Ready-made, convenience foods and composite dishes had the highest proportions of eligible foods in school canteens based on the WHO-EMR model (Table 3). But, cakes, sweet biscuits and pastries had the largest share in permitted foods when the assessment was done based on the healthy nutrition canteen guideline of Iran (Table 3). Also, this group had the largest share of not permitted food groups among food items existed in school canteens under both models (Table 3). The comparison of total permitted/not permitted food items in the schools with different characteristics (Table 4) showed only a significant difference between rural and urban areas by the WHO-EMR model. Permitted foods were found more in the urban school canteens, and conversely, not permitted foods had a larger share in the school canteens of rural areas.

### Policy evaluation

Many interviewees believed that it is necessary to monitor and evaluate the HSC program. Insufficient trained and capable human resources was one of the main limitations for monitoring and evaluation of canteens health status in the schools across the country. Presently there are separate supervisory checklists being used by the offices at the MoHME and MoE which is a redundant and making them as one can save time and resources. Moreover, interviewees stated that the checklists are more focused on the hygienic aspects of the environment and food items, rather than the nutritional value of the foods. Besides, dealing with violators has not been strict enough. Environmental health offices in community health centers are the only sector for dealing with violations legally. However, for them monitoring school canteens was a lower priority, compared to other tasks they are responsible for.

*"We are environmental health experts* [Inspectors]*. Our priority is to supervise restaurants, delicatessen, and food preparation and distribution venues. In fact, there are all kinds of places that need to be inspected and monitored. The school canteen is one of them that we have to visit once every three months. Since it is not possible to visit all schools every three months, if the experts* [Inspectors] *have time, do it …* [But] *This schedule is idealistic and experts* [Inspectors] *cannot implement it*.*"* (A representative from the Environmental Health of Deputy of Health in one of the Universities of Medical Sciences)

Therefore, it is necessary to consider sufficient human resources supply to monitor the school canteens regularly.

Another challenge in monitoring and evaluation of the program was the lack of an official report on the results of the program audits. Many interviewees stated that although annual auditing forms are completed in the province level and sent to the national level of the MoHME and MoE, so far no official report on results of the audits has been published. This default has caused stakeholders to disagree about the policy outcome(s).

*“I think the situation of schools’ canteens is wretched. … Health and standard conditions are problematic based on the current criteria. We have few schools where the rules are actually followed”* (A representative of Environmental Health office in the MoHME)

*"In the meetings that we had with the working groups in Tehran … based on the feedbacks and reports about the number of inspections and health status* [of canteens] *… . every step that we move forward it has improved … "* (A representative of Health office in the MoE)

To avoid any misunderstanding, it is necessary to publish official reports on the current situation on a regular basis. In addition, based on the interviewees no evaluation of the program has been carried out since its implementation. This has prevented policy makers and other actors to access objective evidence for taking decision(s) on changes required to improve the content and implementation of the program.

## Discussion

The findings of the current study revealed that four years after implementation of the policy of healthy school canteens in Iran, more than 54% of available foods in school canteens of Tehran did not comply with the list of permitted foods of the national guideline. This figure was more than 85% when compared with the WHO-EMR recommendations for children. These findings indicate a higher rigidity of the WHO-EMR model compared to the HSC guideline. The most important reason is inadequate consideration to scientific, accurate, and transparent criteria in the HSC guideline. The cakes, sweet biscuits, and pastries group had the highest share among the food groups available in the school canteens. Although all types of food items belong to this group are prohibited in the WHO-EMR model, but all of them were allowed in the current HSC guideline during the study period, except for types of donuts and non-meat strudel without packaging and no hygiene licensed from the MoHME.

The proportion of not permitted food items was significantly higher than permitted items based on both models. Cakes, sweet biscuits and pastries had the highest share among not permitted foods. Also, these food items have a large share of processed food products that Iranian children consume on a daily basis [58]. A cross-sectional study in Kerman, a province in central Iran, also pointed to the low compliance of food products available in school buffets with national guideline [40]. Despite these facts, key informants interviewed believed that the school canteens have improved compared to before the program was initiated.

There are similar experiences in many countries, e.g., Pakistan, India, Bahrain, Saudi Arabia, Oman, Lebanon, Egypt, Turkey, where foods sold in school canteens had low nutritional value and contained items high in sugar (cakes, biscuits, confectionaries, sugary drinks), fat (French fries, fried foods), or salt (crackers, chips) [59–63]. The deliciousness of unhealthy foods, the high profitability of selling them, and inadequate monitoring are considered the main culprits behind such outcomes [59–61, 63]. However, in some neighboring countries, such as Pakistan, there are no guidelines for school buffets [63]; the food-based guideline for school canteens in many Middle Eastern countries, e.g., Iran has concentrated on hygienic conditions and food safety, and there is a poor focus on the nutritional value of the foods [62]. Furthermore, in Iran, like many other LMICs, there is a limited number of healthy food options, and their high price has been recognized as a barrier to implementing HSC policy [64].

Changing the food environment to offer healthier foods is not easy [65]. Several studies have reported that not permitted foods are sold at schools even after the implementation of health canteen policies [33, 66–70]. A review of school food policies in eight countries in Latin America indicated high availability of nutritionally poor foods in kiosks and out of schools, while in seven of these eight countries, there was a legislation regarding the sale of foods in school shops/kiosks [54]. For instance, Mexico has taken action in recent years to reduce the availability of unhealthy foods in schools, but it continues to face challenges. Food and beverage guidelines for elementary schools was established by the government in 2010 [3]. However, a study of 39 schools in 2017, showed that energy-dense foods banned in the guidelines were still widely available, while plain water, fruits and vegetables, accounted for less than 7% of the foods and drinks available in schools [71].

Also, in higher-income countries, while provision of nutritious foods or standards to restrict the availability of foods and beverages high in energy, sugar, salt, and fat have had a positive influence on dietary behaviors [72], in Australia, an evaluation of the policy guidelines to promote nutritious food sales in school canteens have shown unsatisfactory results [73].

In the present study, comparing the proportion of permitted and not permitted food items based on the WHO-EMR model did not show a significant difference in public/private, girls’ /boys’ schools and in different districts of Tehran province; however, the situation in rural schools was worse than urban ones. Other studies showed that the level of obedience to the policies change by socio-demographic characteristics of schools [69], type of the policies (e.g., nutrient-based versus food-based) [33, 68, 69], coexistence of state and national-level of policies [74], and years that schools adopted the policies [69].

One of the questions the present study explored was why school canteens still supply and sell the unauthorized foods despite the national guideline. Based on the results, school canteen managers, as front-line implementers are mostly janitors and often do not follow the law thoroughly. One of the reasons for their reluctance in the implementation of the law is the financial benefits of selling unhealthy food. For achieving high levels of health in food canteens, it is necessary that the school principals have a clear and strong authority, with a pure commitment to healthy food on school environments. However, the findings showed that this is not the case for some principals and they collaborate in making money through selling more of unhealthy products. In addition, low diversity and high price of healthy foods in the market and children’s preferences towards sweet, fatty and salty foods are factors that limit the full implementation of the law. After all, dealing with the offenders by both school officials and health inspectors had not been strict enough and the policy has not been taken seriously. Over all, barriers in school nutrition policy implementation can be a combination of complexity of policies, students’ food preference, fears of revenue loss, limited resources, and a lack of coordination, support, and communication [32, 75–77].

One of the main weaknesses of the HSC policy in Iran is applying a top-down approach in the formulation and implementation of the program that may have unpleasantly affected accountability and ownership of actions [78]. Failure to use clear and measureable criteria (e.g. the maximum amount of sugar, salt, or fat in 100 g of products) to determine the recommended food list have created ambiguity and inconsistency in the judgment regarding the appropriateness of some foods and beverages being included in the guideline. The results of the present study are in-line with the findings of a qualitative study on the Philippines’ school food policies that indicated different perceptions of policies had led to different interpretations of the permissibility of certain foods in schools [22].

Today, many countries use the nutrient content (e.g. energy, fats, sugars or salt/sodium) to set forth standards, guidelines or regulations for foods and beverages available in schools. In this regards, countries in the WHO South-East Asia Region have the most comprehensive inclusion of nutrients in their criteria [79]. Globally, criteria based on energy or salt/sodium content, or both are more common, while in the Eastern Mediterranean region of the WHO, the most common criteria used are based on sugar content [79].

In the formulation of HSC policy in Iran, individual, socio-cultural and physical-environmental influences have not been addressed properly, while these factors have been shown to affect unhealthy snacks consumption among adolescents in the country [40]. Also, not involving all stakeholders in the formulation phase of the policy, has resulted in skepticism and resistance from some of them, including food producers during the implementation phase. Monterrosa showed that food industry believed that the school food guidelines was discriminatory against processed foods and there was insufficient evidence to support that processed foods and beverages are the cause of obesity problem and children should educate how to choose food rather than have a good eating behaviors through restriction and prohibition [29]. Therefore, building commitment and consensus among many stakeholders is essential for successful implementation and development of policies [80].

Other barriers identified in the implementation of HSC program in Iran, included limited financial and human resources (e.g. lack of health coaches in some schools), high workload of key actors (e.g. school principals and health inspectors in community health centers), and poor monitoring systems. Similarly, Reeve et al. reported that despite the existence a strong policy mechanism to provide healthy food in the Philippines schools, limited human and financial resources for its planning and implementation, negatively affected the policy impact on the health of school food environment [22].

### Strengths and limitations

To the best of our knowledge, the present study is the first analysis on school food policy in Iran. The strengths of this study lie in its stringent methodology. A depth policy analysis was performed by a combination of quantitative and qualitative data through a mixed method, and triangulation theory.

There are some limitations that should be considered in interpretation of the current results. The interviewees might have responded with regard to their societal, political, and institutional commitments and tried to manner socially desirable, which could affect the information they provided. Another limitation is the recall bias that we tried to minimize through adding document content analysis. Moreover, in some schools, it seemed that some of the food items were hidden before evaluating food items available in the canteen. Therefore, data collection may have been associated with some bias.

### Policy recommendations

Based on the findings, the following actions are suggested. It is necessary for decision-makers to use an evidence-based approach to policy making and choosing food items for provision in school canteens based on precise and scientific criteria and recommendations. In this regard, using a regional tool, e.g., the Nutrient Profile model of the WHO-EMR [51], may be beneficial. In addition, it is essential to adopt proper economic policies to increase access to healthy food items, with special emphasis on vulnerable and low-income groups, e.g., through subsidies. All policy actors should be given opportunities to state their opinions on the policy and involve in the policy process. Active and effective interaction between actors will lead to shared understanding and awareness of the policy. One of the key factors in the policy success is improving inter-sectoral collaboration between the health and education sectors, the media, principles schools, parents, food producers and food industries, private sectors, local agricultures. The food industries must be justified to increase more socially accountable about children s’ health and encouraged by suitable incentives policies to produce healthy food items for use in children’s food environments. Practical programs should be formulated for easy access to fresh fruit and vegetables in schools. Involving locale agriculture to provide these foods or adopting school gardens/greenhouses strategies can be useful. Non-governmental organizations (NGOs), and benefactors can also be helpful in providing healthy food packages at reasonable prices for children in deprived areas. In addition, private sectors can help to build/reconstruct standard school canteens or provide the required equipment. It is also necessary to consider strategies for regular, systematic monitoring, continuous policy evaluation, documenting evidence, and accessible reporting. Involving the academic sectors to create evidence and suggest the best policy options can be very helpful. The availability of safe, nutritious, and enjoyable food, supported by the HSC policy, accompanied by nutrition and food education, accelerates and supports better food choices and practices in the school setting.

## Conclusion

The current findings indicate that the HSC policy has not been fully implemented in primary schools in Iran. The adoption of bylaws and regulations to protect children from the adverse effects of unhealthy foods and beverages in the school setting was restricted by the capacities and resource shortages for implementation, poor intra/inter-sectoral partnership, and potential conflicts of interest with food companies and canteen managers. The findings provide valuable information for policy makers, school officials, and public health practitioners interested in supporting the health and nutrition of children.

## Supplementary Information

**Additional file 1.**

**Additional file 2.**

**Additional file 3.**

## Data Availability

The datasets generated and analyzed during the present study are availablefrom the corresponding author on reasonable request.

## Abbreviations

WHO: World Health Organization
WHO-EMR: World Health Organization-the Eastern Mediterranean Region
HSC: Healthy School Canteen
MoHME: Ministry of Health and Medical Education
MoE: Ministry of Education
IRAN-ECHO: IRAN-Ending Childhood Obesity
