# Supplementary material for: Scrutinize of healthy school canteen policy in Iran’s primary schools: a mixed method study
Source: BMC Public Health. 2021 Aug 18;21:1566. doi: 10.1186/s12889-021-11587-x (PMC8375065; doi:10.1186/s12889-021-11587-x)
Supplement: Supplementary file 1 — Additional file 1. [file 12889_2021_11587_MOESM1_ESM.docx]

Additional file 1

**List of questions used in semi-structured interviews with key informants from healthy school canteen policy in Iran**

M / F

To start with, could you just tell me a little bit about who you are? )Profession / organization(

Your name and affiliation are completely confidential and will not be published anywhere. You can also not answer a question at your discretion, or interrupt the interview.

1- What do you know about the history of healthy school canteen policy in Iran?

2- What was the purpose of this policy? What effect can the implementation of this policy have on the nutritional status of children?

3- What are the key contextual factors and events that move the healthy school canteen policy to the agenda setting?

4- Who are the main stakeholders involved in each different stages of the policy cycle?

5- What are the challenges faced and opportunities followed during healthy school canteen policy in Iran (in all stages of policy cycle)?

6- What is your opinion about implementation of this policy?

7- Have you seen the current list of permitted/not permitted food items offered for the school canteen? Do you agree with all that? Is there any food item you would like to remove or add?

8- Would you please, give your opinion about the criteria to regular the list of permitted/not permitted food items for school canteens?

9- Please, explain the strengths and weaknesses of the policies?

10- What other key informant do you suggest to be interviewed in this regard?

11- As a final question, is there any other ideas or comments about healthy school canteen policy in Iran you would like to add?
