# Supplementary material for: Scrutinize of healthy school canteen policy in Iran’s primary schools: a mixed method study
Source: BMC Public Health. 2021 Aug 18;21:1566. doi: 10.1186/s12889-021-11587-x (PMC8375065; doi:10.1186/s12889-021-11587-x)
Supplement: Supplementary file 2 — Additional file 2. [file 12889_2021_11587_MOESM2_ESM.docx]

Additional file 2

**School Information Collection Form**

| School name | |  | | |
| --- | --- | --- | --- | --- |
| Address | |  | | |
| Tell | |  | | |
| Number of students | |  | | |
| Type of school | | Public | Private | |
| Gender of students | | Girl | Boy | |
| Education area |  | | | |
| Is school a health promoting school? | | | Yes | No |
| Are there any rules for permitted/ not permitted foods in the canteen?  Yes No  If yes, list of permitted food items  ……………………………………………………………………………………………..  List of not permitted food items  …………………………………………………………………………………………….. | | | | |
