# Supplementary material for: Scrutinize of healthy school canteen policy in Iran’s primary schools: a mixed method study
Source: BMC Public Health. 2021 Aug 18;21:1566. doi: 10.1186/s12889-021-11587-x (PMC8375065; doi:10.1186/s12889-021-11587-x)
Supplement: Supplementary file 3 — Additional file 3. [file 12889_2021_11587_MOESM3_ESM.docx]

**Good Reporting of a Mixed Methods Study (GRAMMS) checklist**

| **Guideline** | **Section: page** |
| --- | --- |
| Describe the justification for using a mixed methods approach to the research question | Background: 6-7  Methods:7 |
| Describe the design in terms of the purpose, priority and sequence of methods | Methods: 7 |
| Describe each method in terms of sampling, data collection and analysis | Methods: 7-12 |
| Describe where integration has occurred, how it has occurred and who has participated in it | Methods: 7-8  Results: 22-27 |
| Describe any limitation of one method associated with the present of the other method | Discussion: 34 |
| Describe any insights gained from mixing or integrating methods | Discussion: 29-33 |

O'Cathain A, Murphy E, Nicholl J. The quality of mixed methods studies in health services research. J Health Serv Res Policy. 2008;13: 92-98.
